# Unusual seeding mechanism for enhanced performance in solid-phase magnetic extraction of Rare Earth Elements

**DOI:** 10.1038/srep43740

**Published:** 2017-03-07

**Authors:** Elizabeth Polido Legaria, Joao Rocha, Cheuk-Wai Tai, Vadim G. Kessler, Gulaim A. Seisenbaeva

**Affiliations:** 1 Department of Chemistry and Biotechnology, Department of Molecular Sciences, Swedish University of Agricultural Sciences (SLU), Box 7015, 75007 Uppsala, Sweden; 2 Department of Chemistry CICECO, University of Aveiro, Aveiro, Portugal; 3 Department of Materials and Environmental Chemistry, Stockholm University, 10691 Stockholm, Sweden

## Abstract

Due to the increasing demand of Rare Earth Elements (REE or RE), new and more efficient techniques for their extraction are necessary, suitable for both mining and recycling processes. Current techniques such as solvent extraction or solid adsorbents entail drawbacks such as using big volumes of harmful solvents or limited capacity. Hybrid nanoadsorbents based on SiO_2_ and highly stable γ-Fe_2_O_3_-SiO_2_ nanoparticles, proved recently to be very attractive for adsorption of REE, yet not being the absolute key to solve the problem. In the present work, we introduce a highly appealing new approach in which the nanoparticles, rather than behaving as adsorbent materials, perform as inducers of crystallization for the REE in the form of hydroxides, allowing their facile and practically total removal from solution. This induced crystallization is achieved by tuning the pH, offering an uptake efficiency more than 20 times higher than previously reported (up to 900 mg RE^3+^/g vs. 40 mg RE^3+^/g). The obtained phases were characterized by SEM-EDS, TEM, STEM and EFTEM and ^13^C and ^29^Si solid state NMR. Magnetic studies showed that the materials possessed enough magnetic properties to be easily removed by a magnet, opening ways for an efficient and industrially applicable separation technique.

Rare Earth Elements (REE or RE) have since 2010 been declared as critical raw materials for many emerging technology applications by the European Commission and the U.S. Department of Energy^1^,^2^,^3^,^4^,^5^. REEs are essential for many high-tech applications that in smaller or larger extent constitute the basis of our life the way we nowadays envisage it. Among the most common applications are wind turbines, NiMH batteries, hard disk drives, phosphors in colour television and flat panel displays, high field strength magnets, electro-optical devices, rechargeable batteries for hybrid and electric vehicles and numerous medical devices^1^,^2^,^3^,^6^,^7^,^8^. An application that recently has received a special attention is creation of REE-doped photoluminescent materials, for application in up-conversion based new light and imaging sources^9^,^10^,^11^. This has logically led to an increase in the demand for these materials and consequently, an effort to ensure a sustainable REE raw materials industry in Europe has been made, involving mining, extraction from wastes or recycling.

Primary mining brings along some intrinsic issues though. Mining comprises currently the majority of the acquisition of REE, and like most mining operations results in large quantities of unwanted excess secondary materials, or even other REE that are less requested or valuable^12^. For example, among light rare earth elements (LREE), Neodymium is one of the most important ones in terms of usage and applicability (it is essential for NdFeB magnets), so the supply of sufficient amount of neodymium to meet its demand, implicates an excess of other metals which are far more abundant like Lanthanum or Cerium. This is known as the “balance problem”^13^. Moreover, mining and processing operations always involve potential environmental risks to human health and to the environment. When it comes to recycling, the balance problem is overcome, and also the composition of the obtained mixture is much simpler, but problems arise from the difficulty of successfully dissolving and selectively extracting the REE.

Currently, most of the efforts in development of REE extraction from solutions are directed towards solvent extraction, in particular, with ionic liquids or the use of ion-exchange resins. Solvent extraction is industrially the most extensively used separation technique at present^14^,^15^,^16^,^17^,^18^,^19^ and although it is possible to accomplish rather high extraction and separation factors, it is associated with considerable challenges such as the need in big volumes of extractant in high concentrations, which translates into elevated costs and environmental hazards. Ionic liquids (IL) have been also intensively investigated^20^,^21^,^22^,^23^ and appeared as a promising and environmentally friendlier alternative to compared to conventional solvent extraction. However, they usually require operation at elevated temperatures (which results in high costs), and some of them, for instance fluorinated IL, being harmful compounds both for human health and environment. The use of IL is facing also problems of recyclability, which may additionally increase the costs. Ion-exchange resins are a cheaper and greener alternative^24^,^25^,^26^,^27^,^28^,^29^, but often have selectivity problems, leading to parallel uptake of other cations such as iron might interfere and foul the resin. Another intrinsic drawback of resins is their relatively quick aging.

As for solid inorganic adsorbent technologies, hybrid nanoadsorbents have been widely studied as very interesting and potentially efficient materials. A special focus has been set to the development of SiO_2_ nanoparticles^30^,^31^,^32^,^33^. Due to its small-diameter particles, ultrafine nanoscale silica offers many advantages for adsorption^34^. When comparing with bulk material, SiO_2_ nanoparticles exhibit much higher surface area and can be surface-functionalized with organic molecules, resulting in very interesting materials for adsorption of REE. In recent works, we developed custom-produced SiO_2_ nanoparticles surface functionalized with three different organosilane derivates^35^. These hybrid nanoadsorbents showed a quick and quite reasonable uptake capacity towards RE^3+^ cations in solution and could even act as luminescent probes for the presence of REE. For industrial applications, the adsorbent needs to be easily removable. This can be achieved using magnetic silica based nanoadsorbents, recently reported by our group^36^. It has been demonstrated that solid inorganic or hybrid nanoadsorbents were reasonably efficient for REE adsorption, being comparable to, for example, ion-exchange resins at neutral pH^24^,^28^.

The adsorption efficiency, however, is limited by the amount of organic ligand that it is possible to graft onto the surface of the inorganic material. The adsorption of REE was in this case achieved by complexation solely via carboxylate groups, while the amino functions remained protonated^37^.

The majority of structurally characterized complexes of REE with amino carboxylate ligands, however, have been produced at elevated pH (in the range 8.5–10), featuring chelation with involvement of amino groups into complexation^38^,^39^,^40^. It is thus surprising that very little has been studied so far about adsorption by nanomaterials under different pH conditions. Gładysz-Płaska and co-workers^41^ studied the adsorption and desorption of lanthanides on red clay, varying pH from 3 to 9 on the desorption process, and Gavarini and co-workers^42^ studied the influence of pH in the process of dissolution of lanthanides from glass in aqueous media. Tuning of experimental parameters in adsorption such as pH or temperature might be the key for an improvement.

In the present work, we turned to investigate the adsorption of REE cations by magnetic silica nanomaterials at elevated pH, looking for the possibility to improve the uptake capacity. Quite unexpectedly, the effect turned to be much more pronounced than it could be anticipated, revealing a different and much more attractive uptake mechanism.

## Results and Discussion

### REE uptake mechanism by hybrid adsorbents

**Figure 1:**
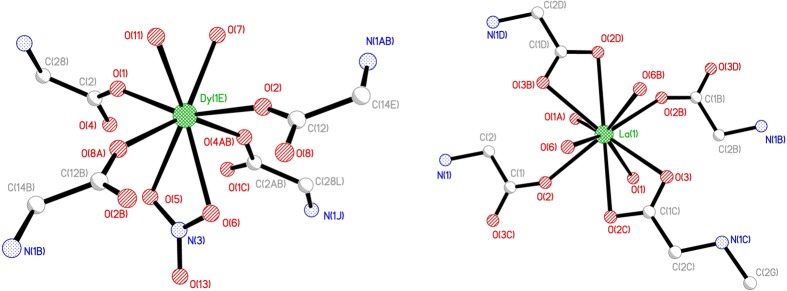
Molecular models of Dysprosium and Lanthanum surface complexes with iminodiacetic acid at neutral pH (from X-ray single crystal data 36).

The original focus of this work was set on providing a deeper insight into the mechanistic aspects of surface functionalization of the nanoadsorbents and optimizing their interaction with REE cations in solution. In our previous study^36^, we investigated the interaction between an iminodiacetic acid organosilane derivate attached onto the surface of core-shell magnetic nanoparticles and REE cations in solution. Our initial hypothesis was that REE cations should chelate with the organic ligand, assuming that the nitrogen atoms would be involved in the chelation via their free electron pair. However, X-ray crystallography performed on model compounds with 1:1 metal-to-ligand ratio as observed in the surface complexes (Fig. 1) showed that the real mechanism did not involve the nitrogen atoms but was governed via concerted action of the carboxylic groups.

It turned out that the adsorption conditions were too acidic for this ligand and the nitrogen atoms were protonated, therefore not being able to contribute to the coordination mechanism.

An apparent step to improve the efficiency of this type of materials was to modify the conditions of adsorption, opening the possibility for N atoms to act in coordination, thus increasing the uptake of REE. Therefore, we performed the same type of experiment varying the pH conditions and creating a basic environment for adsorption. The uptake efficiency turned hugely enhanced, reaching maximum values of up to 440 mg REE^3+^/g and 900 mg REE^3+^/g for SiO_2_ and SiO_2_ covered magnetic nanoparticles, respectively. This was in fact totally unexpected and indicated a completely different way of action for the adsorbent.

### Enhanced uptake efficiency of REE cations by nano adsorbents under basic conditions

The materials were analysed after loading of REE by energy-dispersive X-ray spectrosctopy (EDS) on the surface of the nanoadsorbents or by complexometric titration. A number of materials were selected to be tested by the latter method, which gives a more exact value of the amount of REE uptaken by the nanoparticles. It requires prior neutralization of all the solutions before performing the titration, therefore a group of representative materials bearing neodymium were selected for this procedure. As it can be observed in Table 1, the high uptake observed by EDS analysis is confirmed by complexometric titration. In the same molar ratio REE: ligand as previously reported for neutral conditions^36^, the observed capacity was almost three times higher, and therefore on a logical further step, the initial concentration of Nd was increased in order to check if this lead to an improved efficiency. Indeed the uptake could be more than 20 times higher than what was observed at neutral pH, where the mechanism was governed by concerted action between the terminal carboxylic groups of the ligand and the REE cations. This huge increase indicated that in this case, the mechanism should be different. Therefore, we investigated whether the presence of complexing ligand on the surface of the nanoparticles was needed or could be avoided. For this matter, both bare SiO_2_ and core-shell γ-Fe_2_O_3_-SiO_2_ nanoparticles were tested under the same procedure, showing indeed that there was no need for a functionalized surface. In fact, the non-functionalized nanoparticles showed actually a higher uptake than the surface decorated ones.

Due to the fact that EDS is a surface and local analysis, at least 5 different and random areas of the surface were chosen and the average values were calculated. Supplementary Table S1 shows the adsorption results on different nanoadsorbents tested, where a very impressive uptake could be observed for both materials tested (SiO_2_ nanoparticles and core-shell γ-Fe_2_O_3_-SiO_2_ nanoparticles), achieving up to 78% of REE in relative weight percentage for Nd in magnetic nanoparticles, and up to 56% and 46% for Dy and La respectively in SiO_2_ nanoparticles.

### Stability of the materials

#### Consecutive adsorption-desorption cycles

One of the main goals of this technique is to develop a new material which can be consecutively used in several adsorption-desorption cycles, and therefore this technique was tested on core-shell magnetic γ-Fe_2_O_3_-SiO_2_ nanoparticles for Dy^3+^ solution in up to three consecutive adsorption-desorption cycles with a simple 24 hours acidic desorption process.

Table S2 shows the EDS analysis of the nanoparticles after the various adsorption or desorption processes, showing that the material is totally reusable, achieving high uptake values (up to 87%) and almost total release of the lanthanide in the desorption process with equal or less than 0.5% of Dy^3+^ remaining on the nanoparticles after the desorption.

### Desorption under less harmful conditions

#### Multi-step desorption with (NH_4_)_2_SO_4_ and acidified (NH_4_)_2_SO_4_ solutions

In the view of potential industrial application of this technique, acidic desorption might not be the most desirable process since it would lead to big amounts of waste acid solutions that would have to be treated before discarding, which translates into an increase in costs and related environmental issues. Therefore, we studied the possibilities of performing desorption under milder conditions, using (NH_4_)_2_SO_4_ as selected desorbing agent or its slightly acidified solution (pH = 3.75). Table 2 shows the summary of the desorption results of the different procedures tested, as determined by complexometric titration. The study was made for magnetic core-shell γ-Fe_2_O_3_-SiO_2_ nanoparticles bearing Dy^3+^, and the results of desorption are given in percentage related to the initial amount adsorbed. As it can be seen from the table, quite competent desorption could be achieved with non-acidic reagents if the process was carried out in three consecutive steps. Most of the Dy^3+^ was released during the first two 3-hours steps, while the last and longest step (20 h) was not really significant for the whole process as it only led to an increase of less than 2%. It is worthy to mention that, whereas EDS analysis showed an almost complete removal of REE from the particles (Table S2), with less than 0.5% of REE after desorption, the results presented in Table 2, where a maximum of 62% is retrieved to the solution, might lead to confusion at first glance. This is due to the fact that REE hydroxides are soluble in strong mineral acids but significantly less soluble in water. For example, crystalline Nd(OH)_3_ solubility in water was found to be 4.8·10^−5^ moles/L^43^, meaning that there is a partial recrystallization of RE(OH)_3_ during the process of neutralization. This recrystallized portion of RE(OH)_3_ is not accessible in solution to complexate with EDTA, being thus not possible to quantify it by complexometry.

### Unravelling the mechanism

#### Interaction of the functional surface layer with REE cations

Solid state ^13^C and ^29^Si cross-polarization magic-angle spinning (CP-MAS) NMR was used initially to investigate the produced ligand-grafted particles and also the same particles after adsorption of non-magnetic La^3+^ cations at pH = 9.

**Figure 2:**
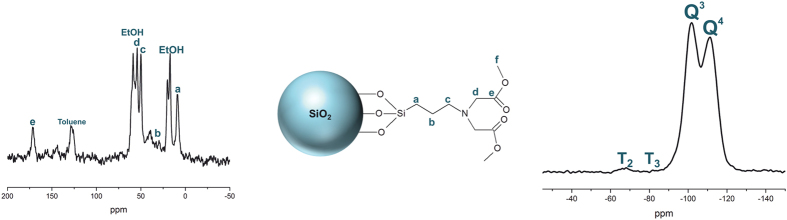
^13^C CP-MAS NMR and ^29^Si CP-MAS NMR spectra of IDA-derivate attached onto the surface of SiO_2_ NPs.

Figure 2 shows the labelling (a to f) of the carbon atoms of the IDA derived ligand molecule and the corresponding ^13^C CP-MAS NMR resonances. The characteristic C = O peak appears at 171.7 ppm. This spectrum also reveals the presence of traces of EtOH and toluene, which were used as solvents in the process of grafting of the ligand. The resonance given by the methyl esther groups (f) of the original siloxane reactant is absent, indicating that, as expected, this function was removed by hydrolysis, catalysed by the surface Si-OH groups, leaving unprotected carboxylic acid functions, essential for complexations. The ^29^Si CP-MAS NMR spectrum exhibits several resonances assigned to Q^n^ and T_n_ local environments^44^,^45^. The peaks at −102 and −110 ppm are ascribed to the silica network (Q^3^ and Q^4^ respectively). Weaker peaks attributed to T_3_ and T_2_ environments can be observed at −80 and −68 ppm respectively.

**Figure 3:**
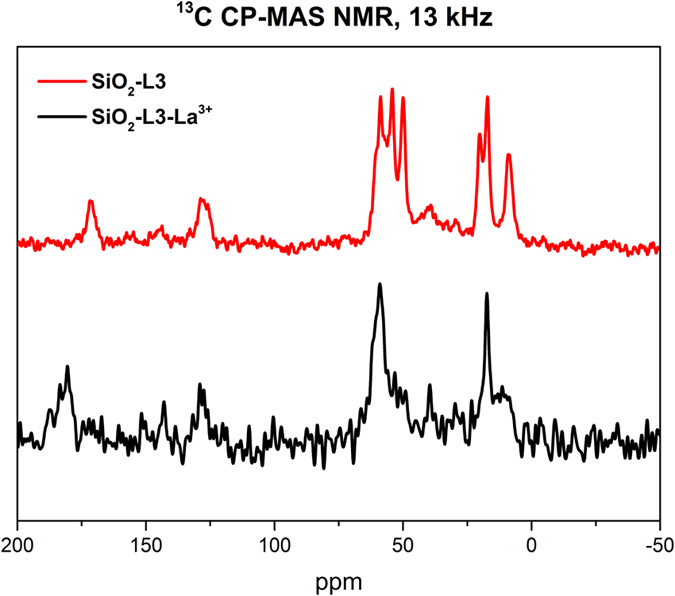
^13^C CP-MAS NMR spectra of surface functionalized SiO_2_ nanoparticles (top) and the same nanoparticles bearing La^3+^ ions (bottom).

The effect of the adsorption of REE (La^3+^ in this case, as a non-paramagnetic lanthanide is suitable for NMR) on the surface of the nanoparticles was investigated by comparing the spectra of the material loaded and unloaded with La^3+^ (Fig. 3). The results provide evidence for the coordination of La^3+^ by the terminal carboxylate groups of the IDA-derivate structure: the carboxylate (C = O) resonance shifts from 171.7 ppm for the unloaded sample to 174.5 ppm for the La^3+^-bearing material.

**Figure 4:**
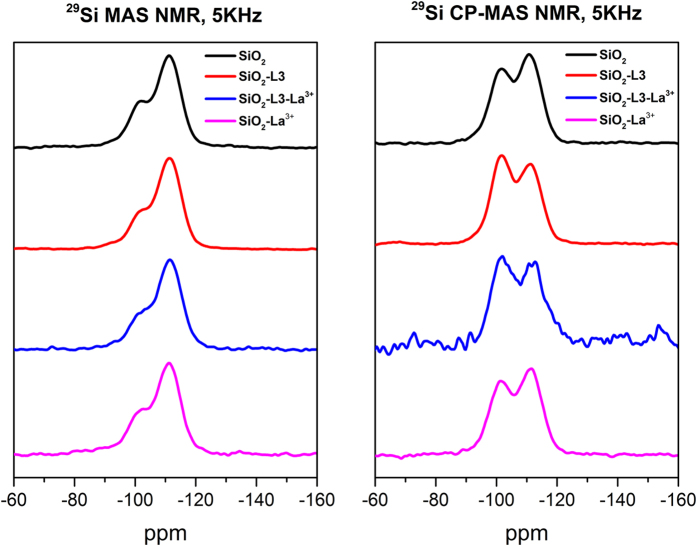
^29^Si MAS and ^29^Si CP-MAS NMR spectra of pristine SiO_2_ NPs (black), surface functionalized SiO_2_ NPs (red), surface functionalized with induced crystallized La^3+^ SiO_2_ NPs (blue), and SiO_2_ NPs with induced crystallized La3^+^ (pink). Every individual spectrum has been normalized to the highest peak.

^29^Si MAS and CP-MAS analyses were also used as a tool to check if the increased pH affected the silica structure. For this, we performed these analyses on pristine SiO_2_ nanoparticles, IDA-derivate surface functionalized SiO_2_ nanoparticles and the latter nanoparticles with induced crystallized La^3+^ (Fig. 4). The ^29^Si MAS NMR spectrum shows a decrease in the number of surface OH (Q^3^) groups when the IDA-derivate ligand is grafted on the surface of SiO_2_ NPs, which constitutes another proof for this process. However, the ^29^Si CP-MAS NMR spectra seem to contradict this conclusion because upon grafting the Q^3^ environments increase relatively to Q^4^ sites (this is also observed for IDA-modified with induced crystallized La). However, the grafting reaction probably also brings about a modification of the remaining NPs surface, increasing the number of surface OH groups. Moreover, care must be taken when considering the evolution of the cross polarization resonance intensities because they depend on the dynamics of this process, which renders it *a priori* non quantitative.

Both ^29^Si MAS and ^29^Si CP-MAS NMR spectra show no significant change in the silica network when crystallization of La^3+^ is induced in the NPs. When the process is performed on IDA-derivate surface functionalized NPs, a decrease in the number of Q^3^ groups is observed, due to the grafting on the surface of the SiO_2_ NPs and the chemical coordination between the IDA molecule and La^3+^ ions. In conclusion, no significant change of the silica network or formation of new phases is observed for both cases, when the crystallization of La^3+^ is initiated by pure SiO_2_ NPs or by surface functionalized SiO_2_ NPs.

As it will be later presented, the presence of the IDA-derivate on the surface of the NPs does not play a role in the initiation process of the REE crystallization, and since it has been demonstrated by solid-state NMR that the structure of silica is not affected, further studies were carried out indistinctly on pristine or surface functionalized nanoparticles.

#### The uptake of REE at increased pH

The expected increased efficiency due to pH elevation was anticipated to possibly double the uptake. It turned out, however, to be more than 20 times higher, indicating the presence of a different mechanism.

**Figure 5:**
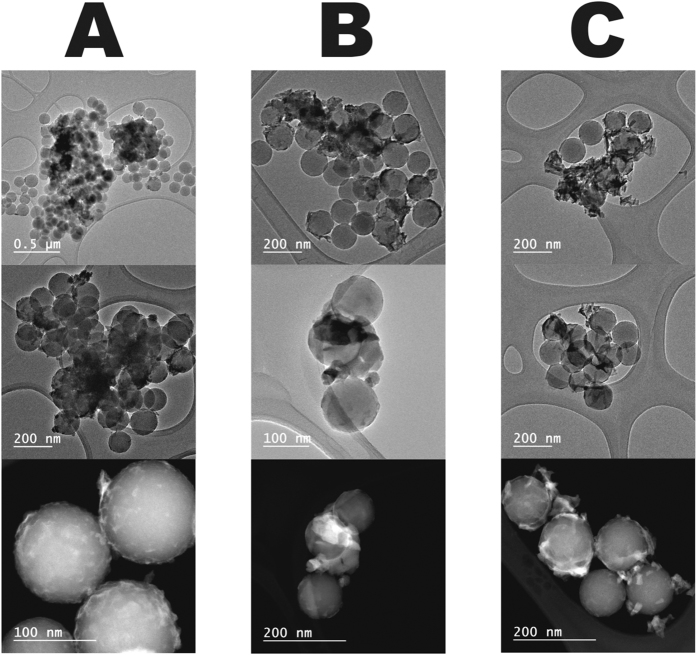
Bright-field TEM (the upper two rows) and HAADF-STEM (lowest row) images of SiO_2_ NPs bearing lanthanides crystallized on its surface at high pH. (**A**) Dy^3+^-bearing particles; (**B**) Nd^3+^-bearing particles; (**C**) La^3+^-bearing particles.

**Figure 6:**
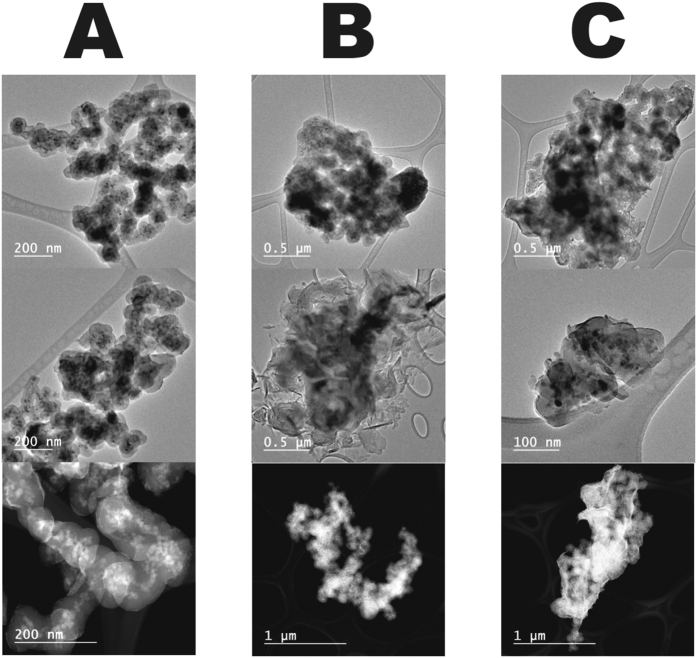
Bright-field TEM (the upper two rows) and HAADF-STEM (lowest row) images of γ-Fe_2_O_3_-SiO_2_ NPs bearing lanthanides crystallized on its surface at high pH. (**A**) Dy^3+^-bearing particles; (**B**) Nd^3+^- bearing particles; (**C**) La^3+^-bearing particles.

**Figure 7:**
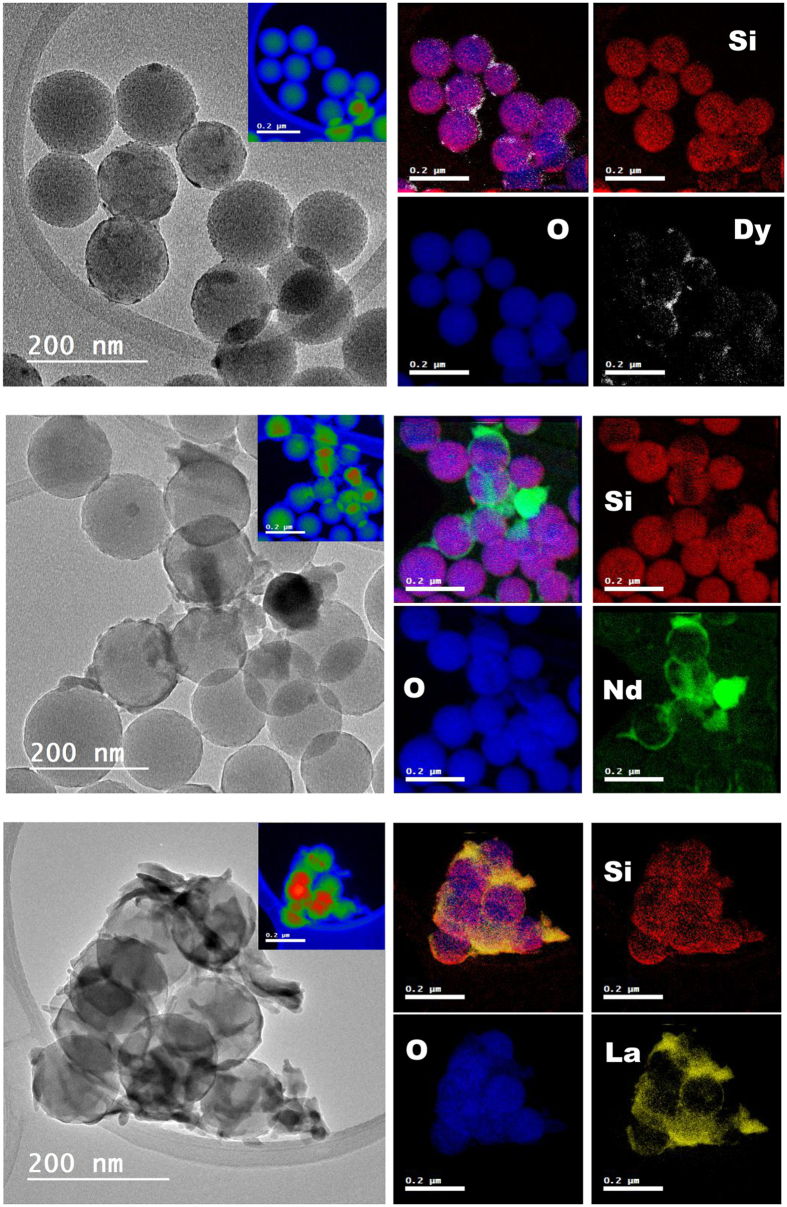
BF-TEM images and elemental mapping by EFTEM of SiO_2_ nanoparticles bearing induced crystallized REE. The insets are the thickness mapping of the regions of interest. The individual EFTEM elemental maps of Si (red), O (blue), Dy (white), Nd (green) and La (yellow) and the overlay of all elements for each sample are shown.

**Figure 8:**
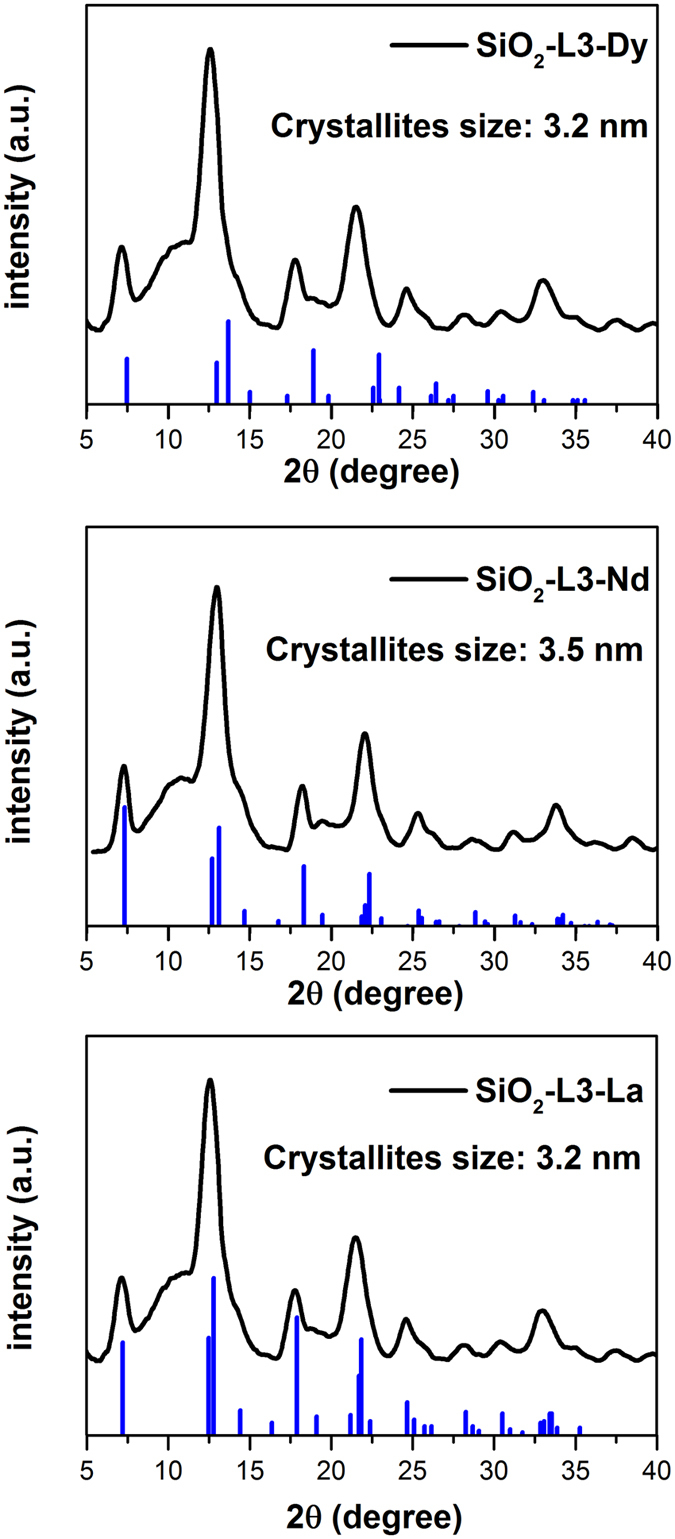
XRPD patterns of IDA derivate functionalized SiO_2_ nanoparticles bearing crystallized REE hydroxides on their surface, the blue lines represent the pattern of the corresponding REE hydroxides, as provided from the crystallographic database included in Bruker EVA-12 software. Crystalline domains sizes were calculated by Debye-Scherrer equation.

In order to understand the involved mechanism, we studied first the morphology of the loaded nanoadsorbents by transmission electron microscopy (TEM) and atomic force microscopy (AFM). Bright-field TEM (BF-TEM) and high-angle annular dark-field scanning transmission electron microscopy (HAADF-STEM) images (Figs 5 and 6) show the formation of a new phase around the surface of the nanoparticles bearing REE. The elemental mapping by EFTEM of SiO_2_ nanoparticles (Fig. 7) confirms the formation of new phases, indicating the formation of a layer of REE compound on the surface of the nanoparticles. Their crystalline nature was revealed by the X-ray powder studies (see Fig. 8). The process did not affect in any significant way the morphology of the nanoparticles, as it can clearly be observed from both BF-TEM and HAADF-STEM images. This indicated clearly that no doping of the original silica phase by the REE ions was taking place in this case, which has also been confirmed by extended X-ray absorption fine structure (EXAFS) studies that did not reveal any other environment in addition to the REE(OH)_3_ hydroxide phase or organic surface complexes, where they were present. It is well demostrated that doping, when it takes place, may lead to a change in the size or shape of the nanoparticles^46^,^47^.

The observed mechanism turned thus to be principally different from the earlier observed selective separation of REE hydroxydes after solubilization of the titanate REE ores via baking them together with alkali hydroxides^48^. The rare-earth oxides selectively formed colloids and floated out away from heavier alkali titanate. In this respect, the proposed and observed mechanism of selective precipitation on magnetic phase is different from that observed in ref. 48.

The driving force of the precipitation of REE hydroxides is apparently the formation of outer sphere complexes between the negatively charged surface of silica at increased pH and the positively charged REE cations. Nucleation of RE(OH)_3_ on the silica surface proceeded as a kinetic phenomenon, resulting in non-uniform growth of this new phase. No Si-O-REE bonds are formed here as this would be associated with extremely large positive Gibbs energy of mixing in such system. No indication for such improbable scenario could be found in the EXAFS spectroscopy data either. The exposed surface of the particles was that of the outer silica layer. No transformation of the crystalline β-Fe_2_O_3_ core of the particles could be observed in the XANES studies of their behavior at different pH^49^.

X-ray powder diffraction (XRPD) of the loaded nanoparticles with the three different REE used (Dy, Nd and La) indicated the formation of isostructural phases of the same nature (Fig. 8).

In all three cases it can clearly be observed that the main characteristic peaks of REE hydroxides are present in the XRD pattern for the loaded SiO_2_ nanoparticles, providing a strong proof of the formation of a crystalline REE hydroxide layer on the surface of the nanoparticles.

**Figure 9:**
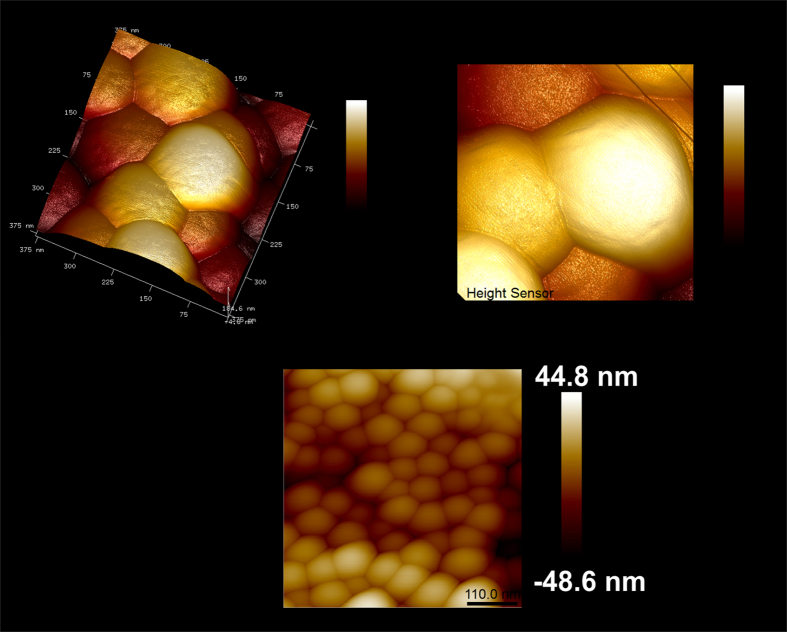
AFM 3D images of Nd^3+^ loaded SiO_2_ nanoparticles (top) and 2D image of pure SiO_2_ nanoparticles.

Figure 9 shows 3D AFM images of SiO_2_ nanoparticles loaded with Nd^3+^, in which it can be noticeable that the deposition of REE(OH)_3_ causes a considerable increase in roughness on the surface of the particles.

Finally, EXAFS spectroscopy was also used as a confirming tool in order to prove the presence of the REE hydroxide layer on the surface of the nanoparticles. For this, we performed EXAFS on surface functionalized SiO_2_ nanoparticles loaded with Dy^3+^.

**Figure 10:**
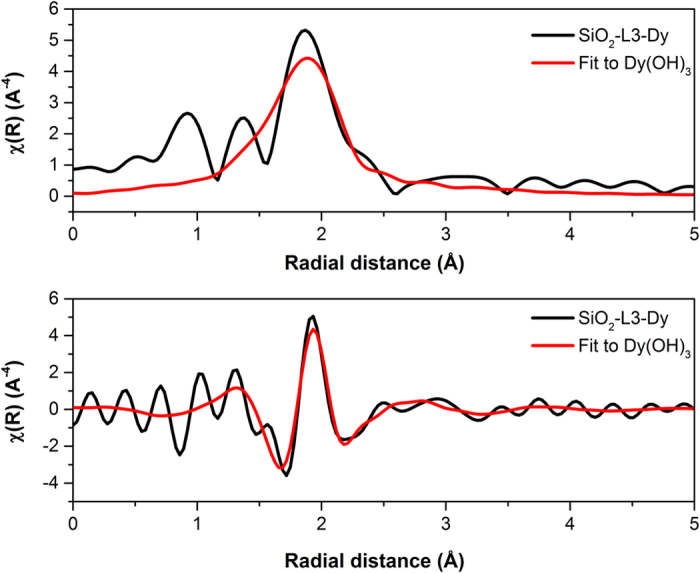
Magnitude (upper) and real part (lower) of χ in R space for the studied SiO_2_ nanoparticles (black) and the fit adjusted to Dy(OH)_3_ structure (red).

Dy(OH)_3_ CIF files were taken from FIZ Karlsruhe ICSD database^50^ and used as reference to fit our collected data using the IFEFFIT program included in DEMETER program package. Figure 10 shows both the original collected data and the data after fitting it to Dy(OH)_3_ structure in R space. The upper graph shows the magnitude and the lower graph shows the real part of chi (χ). Two different single scattering events (Dy-O-Dy, and Dy-H-Dy) and two multiple scattering events (Dy-O-H-Dy and Dy-O-O-Dy) were taken into consideration in the fitting and the r_max_ was limited to 5 Å.

The process of fitting the model structure of Dy(OH)_3_ with our real spectra lead to an R-factor of 0.03 and a χ^2^ of 147.5, which are quite acceptable fitting parameters, and together with the rest of the characterization performed, can give a valid proof of the presence of a layer of Dy(OH)_3_ on the surface of the nanoparticles.

## Conclusion

An exciting and beforehand unexpected new mechanism of seeding of REE onto nanoadsorbents has been envisaged in this work. The initial idea to vary pH conditions in order to involve N atoms of the iminodiacetic acid derivate ligand in the chelation with REE led to a surprisingly enhanced efficiency. It has been proved to occur due to the initiation of crystallization of a layer of RE(OH)_3_ onto the surface of the particles. The optimized uptake, up to 20 times higher than previously observed, was confirmed by complexometric titration and EDS analysis, and the features of the mechanism were explained by solid state NMR, TEM, STEM and EFTEM, powder X-ray diffraction and EXAFS spectroscopy.

This method opens new possibilities for adsorption, since the presence of specific ligands is not crucial, and when the procedure is carried out on magnetic nanoparticles, the material is still easily removable by magnet. In addition, its reusability has been fully confirmed in three adsorption-desorption cycles, demonstrating an easy method highly suitable for industrial applications.

## Methods

### Chemicals

For the nanoparticles synthesis, FeCl_3_ (98%), FeCl_2_ (97%), NH_4_OH (25%), TEOS (98%) and ethanol (99.5%) were purchased from Sigma-Aldrich. For the rare earth adsorption, La(NO_3_)_3_·6H_2_O, Dy(NO_3_)_3_·6H_2_O and Nd(NO_3_)_3_·6H_2_O were purchased From Sigma-Aldrich. NH_4_OH (25%) from Sigma-Aldrich was used to adjust the pH. For desorption experiments, HCl and HNO_3_ were used.

### Synthesis of Fe_3_O_4_ nanoparticles

Magnetite nanoparticles were prepared by co-precipitation of iron (III) and iron (II) chlorides with ammonia in nitrogen atmosphere^51^. The obtained Fe_3_O_4_ NPs were spherical with an average diameter of about 12 nm, according TEM analysis and a specific surface area of about 96 m^2^ g^−1^ 36.

### Synthesis of core-shell SiO_2_ coated γ-Fe_2_O_3_ nanoparticles

Core-shell SiO_2_ covered γ-Fe_2_O_3_ NPs were synthesized via a modified Stöber method leading to highly stable nanoparticles^52^. Following a typical Stöber synthesis, Fe_3_O_4_ nanoparticles (100 mg) were dispersed in miliQ water (32 mL) and sonicated for 20 minutes. Then, the dispersed solution was mixed with ethanol (160 mL) and NH_4_OH (4 mL, 25%) was slowly added. After NH_4_OH addition, TEOS (1.6 mL) was added dropwise, achieving a SiO_2_:FeO_x_ ratio of 6:1. The mixture was stirred for 20 hours. After stirring, the magnetic nanoparticles were separated from the solution with a magnet and then washed three times with dd. H_2_O and twice with ethanol and centrifuged for 10 minutes at 10000 rpm. Finally, the nanoparticles were dried under N_2_ atmosphere.

### Adsorption of the rare earth metals (Dy^3+^, Nd^3+^ and La^3+^) ions

Stock solutions of REE(NO_3_)_3_ 0.02 M, where REE = Dy, Nd, or La were prepared. To 50 mg of core-shell SiO_2_ coated γ-Fe_2_O_3_ nanoparticles, 7 mL of RE salt solutions and 2 mL of NaNO_3_ 1 M were added. Then, a solution of NH_4_OH 5% was added dropwise until the pH of the mixture was around 9. This value was chosen as corresponding to pronouncedly basic medium, where silica is still known to be stable to hydrolysis in aqueous medium^53^. The mixture was shaken in an orbital shaker for 24 hours at 130 rpm. After shaking, the samples were centrifuged at 10000 rpm for 10 minutes to separate the nanoparticles from the solution and washed once with dd. H_2_O (20 mL). Finally, the nanoparticles were dried under N_2_ atmosphere in Schlenk equipment. The detailed results are presented in Table 1.

### Complexometric titrations of lanthanides in mother liquor over SiO_2_ or SiO_2_ coated γ-Fe_2_O_3_ nanoparticles

A first step of neutralization of the mother liquor was necessary. For this, the solution was continuously evaporated and a new portion of dd. H_2_O (20 mL) was added until the pH was neutralized to a value of around 6. After the neutralization, complexometric titration was carried out with EDTA 5 mM using xylenol orange as indicator. EDTA complexates metals in a 1:1 ratio, therefore the amount of RE adsorbed to the surface of the nanoadsorbents by substraction, since the initial amount is known and the remaining amount in solution is determined by this procedure.

### One step desorption experiments in acid conditions for Dy^3+^ ions from the nanoadsorbent

Around 30 mg of nanoadsorbent bearing RE cations adsorbed at high pH conditions were mixed with 5 mL of HNO_3_ or HCl 1 M and 15 mL of miliQ water, obtaining a final mixture with a pH = 1. The mixtures were shake for 24 hours in an orbital shaker. Adsorbent and solution were afterwards separated by centrifugation. The collected solutions were neutralized prior to titration with EDTA. The neutralization procedure carried out was the same as explained before, through evaporation and redilution with miliQ water until pH around 6. After neutralization the RE ions in solution were quantified by titration with EDTA 5 mM as explained before.

### Multi-step desorption experiments for Dy^3+^ ions under milder conditions

A milder and environmentally friendlier procedure for desorption was attempted using (NH_4_)_2_SO_4_ as desorbing agent in several steps. For this, in the first step, around 30 mg of nanoadsorbent bearing RE cations were mixed with 5 mL (NH_4_)_2_SO_4_ 0.5 M and 15 mL miliQ water. This mixture was shaken for a first period of 3 hours. Then, the solution was separated from the nanoadsorbent via centrifugation, and the nanoadsorbent was mixed with a new portion of desorbing mixture (5 mL of (NH_4_)_2_SO_4_ 0.5 M and 15 mL of miliQ water). The mixture was again shaken for another 3 hours and the same procedure was followed until the third desorption step, in which the shaking time was increased to 20 hours. All solutions were collected and the RE ions desorbed were determined by complexometric titration with EDTA 5 mM.

### Multi-step desorption experiments for Dy^3+^ ions under milder conditions at a controlled pH

In order to improve desorption efficiency process under milder conditions, desorption was attempted with a solution of (NH_4_)_2_SO_4_ 0.5 M in which the pH was adjusted to 3.75 via addition of a H_2_SO_4_ 0.5 M solution. The same three-step desorption procedure was carried out as explained before.

### Second and third cycle adsorption-desorption on previously used samples

On the samples in which one step acid desorption was carried out, a second cycle lanthanide adsorption was tested. For this, around 20 mg of the desorbed γ-Fe_2_O_3_@SiO_2_ were mixed with 7 mL of RE(NO_3_)_3_ 0.02 M and 2 mL of NaNO_3_ 1 M. The mixture was shaken on an orbital shaker for 24 hours at 130 rpm. After shaking, the nanoparticles were separated from the solution via magnet and dried under N_2_ atmosphere. Dried nanoparticles were analysed by SEM-EDS and the remaining solution was used for complexometric titration as explained before. The same procedure was followed for a third cycle adsorption-desorption. The detailed results are summarized in Tables 2 and S2.

### Characterization

The ^13^C and ^29^Si solid-state NMR spectra were recorded on a Bruker Avance III 400 spectrometer operating at a magnetic field of 9.4 T at 100.6 MHz and 79.5 MHz respectively. Chemical shifts are quoted in ppm from tetramethylsilane (TMS). ^13^C cross-polarization magic-angle spinning (CP MAS) NMR spectra were recorded on a 4 mm probe with 2.8 μs ^1^H 90° pulses, 2 ms contact time, a recycle delay of 5 s and a spinning rate of 13 kHz. ^29^Si MAS and ^29^Si CP MAS NMR were recorded on a 7 mm probe with a spinning rate of 5 kHz. ^29^Si MAS NMR spectra were collected with an excitation pulse length of 2.7 μs (corresponding to 40° flip angle) and a recycle delay of 60 s. ^29^Si CP MAS NMR spectra were acquired with 3.3 μs ^1^H 90° pulses, 8 ms contact time and a recycle delay of 5 s.

Transmission electron microscopy (TEM), scanning (STEM) and energy-filtered TEM (EFTEM) images were taken on a JEOL JEM-2100F Schottky field-emission microscope operated at 200 kV (C_s_ = 0.5 mm) and equipped with a Gatan Ultrascan 1000 CCD camera, a post-column imaging filter (Gatan Tridiem 863) and a Gatan annular dark-field (ADF) detector. High-angle annular dark-field STEM (HAADF-STEM), also known as Z-contrast, images were acquired in the same microscope with a probe size of 0.2 nm and in HAADF5 mode. EFTEM images were obtained by three-window method to extract the core-loss information. The edge position of each element (Si L-edge, O K-edge and the M_4,5_-edge of La, Nd and Dy) was determined by electron energy loss spectroscopy (EELS) in advance. The results of elemental mapping and thickness variation are presented in pseudo-color.

SEM-EDS studies were performed with a Hitachi TM-1000-μ-DEX scanning electron microscope. For each sample, 5 different points were measured by EDS in area mode (one at x500 magnification, three at x5000 and one at x10000) and the average value was calculated and given as relative content of the elements.

Surface structure was characterized using a Bruker FastScan Bio Atomic Force Microscopy (AFM) operating in tapping mode.

X-ray powder diffraction (XRD) experiments were carried out with a multipurpose Bruker SMART Apex-II instrument. The background substraction and the identification of the patterns were made using Bruker EVA-12 software. The size of the crystalline domains was calculated according to Debye-Scherrer equation (Equation 1):


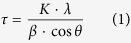


where τ is the size of the crystalline domains, K is a dimensionless shape factor with a typical value of about 0.9, λ is the X-ray wavelength, which for the instrument used is 0.71073 Å, β is the line broadening at half maximum intensity (FWMH) and θ is the Bragg angle.

EXAFS spectra were recorded at the wiggler beam line I811, MAX IV Laboratory, Lund, Sweden. The station is equipped with a Si (111) double crystal monochromator. EXAFS data collection was performed in fluorescence mode and DEMETER software was used for the data treatment.

## Additional Information

**How to cite this article**: Polido Legaria, E. *et al*. Unusual seeding mechanism for enhanced performance in solid-phase magnetic extraction of Rare Earth Elements. *Sci. Rep.* **7**, 43740; doi: 10.1038/srep43740 (2017).

**Publisher's note:** Springer Nature remains neutral with regard to jurisdictional claims in published maps and institutional affiliations.

## Supplementary Material

Supplementary Information

## Figures and Tables

**Table 1:** Uptake of Nd^3+^ in different materials and molar ratios as determined by complexometric titration.

Sample	Molar ratio REE:ligand	pH	Uptake capacity (mg Nd^3+^/g)
**γ-Fe**_**2**_**O**_**3**_**-SiO**_**2**_**-L3**	1:1	neutral	33.6
**γ-Fe**_**2**_**O**_**3**_**-SiO**_**2**_**-L3**	1:1	9	90
**γ-Fe**_**2**_**O**_**3**_**-SiO**_**2**_**-L3**	5:1	9	446.3
**γ-Fe**_**2**_**O**_**3**_**-SiO**_**2**_**-L3**	10:1	9	907.7
**γ-Fe**_**2**_**O**_**3**_**-SiO**_**2**_	5:1	9	861.6
**SiO**_**2**_	5:1	9	442.8

**Table 2:** Desorption as determined by complexometric titration.

Desorption method	Adsorbed (mmol Dy^3+^/g)	Desorbed at first 3 h (%)	Desorbed at 3 + 3 h (%)	Desorbed at 3 + 3 + 20 h (%)	Total desorbed (%)	Desorbed in one 24 h. step (%)
(NH_4_)_2_SO_4_ 0.5 M	3.4	20.1	39.9	2	62	
Acidified (NH_4_)_2_SO_4_ 0.5 M (pH = 3.75)	3.4	23	30.2	1.8	55	
HCl 1 M	3.4	—	—	—	—	51.1
HNO_3_ 1 M	3.4	—	—	—	—	40.9
