# Supplementary material for: Unusual seeding mechanism for enhanced performance in solid-phase magnetic extraction of Rare Earth Elements
Source: Sci Rep. 2017 Mar 7;7:43740. doi: 10.1038/srep43740 (PMC5339825; doi:10.1038/srep43740)
Supplement: Supplementary Information [file srep43740-s1.pdf]

Supplementary Information

Unusual seeding mechanism for enhanced performance in solid-phase magnetic extraction of Rare Earth Elements.

Elizabeth Polido Legaria*, Joao Rocha, Cheuk-Wai Tai, Vadim G. Kessler, Gulaim A. Seisenbaeva.

Supplementary Table S1. EDS analysis on different nanoadsorbents loaded with REE under basic conditions.

Sample	%Al (from the sample holder)	%Fe	%Si	%Dy	%Nd	%La
**SiO_2-La**	-	-	53,8	-	-	46,2
**SiO_2-Nd**	-	-	62,6	-	37,4	-
**SiO_2-Dy**	-	-	44,1	55,9	-	-
**γ-Fe_2O_3-SiO_2-Nd (5x)**	4,1	15,6	16,8	-	63,7	-
**γ-Fe_2O_3-SiO_2-Nd (10x)**	4	8,6	14,5	-	72,8	-

Supplementary Table S2. EDS analysis on three consecutive adsorption-desorption cycles with Dy^{3+} on γ-Fe_2O_3-SiO_2 NPs.

Process	%Fe	%Si	%Dy
**1st cycle adsorption**	24,5	14,2	61,3
**1st cycle desorption**	64,5	32,6	0,5
**2nd cycle adsorption**	16,9	5,7	77,5
**2nd cycle desorption**	71,7	28,3	0
**3rd cycle adsorption**	10,9	2,5	86,9
**3rd cycle desorption**	70,3	29,4	0,3
